# Risk prediction models for postherpetic neuralgia: a systematic review and meta-analysis

**DOI:** 10.3389/fneur.2026.1842982

**Published:** 2026-07-14

**Authors:** Qian Li, Hui Li, Zhejin Yuan, Dongmei Yuan

**Affiliations:** 1 Department of Pain Management, West China Hospital, Sichuan University, Chengdu, Sichuan, China; 2 West China School of Nursing, Sichuan University, Chengdu, Sichuan, China

**Keywords:** herpes zoster, meta, postherpetic neuralgia, risk prediction model, systematic review

## Abstract

**Objective:**

This study conducted a systematic review and meta-analysis of risk prediction models for postherpetic neuralgia (PHN), aiming to provide a reference for Chinese scholars to develop higher-quality risk prediction models.

**Methods:**

This study systematically searched the China National Knowledge Infrastructure (CNKI), Wanfang Data Knowledge Service Platform, VIP Chinese Science and Technology Journal Database, Chinese Biomedical Literature Database (CBM), PubMed, Web of Science, Embase, and Cochrane Library for studies on risk prediction models for postherpetic neuralgia. The search period for all databases was from inception to March 1, 2026. Two researchers independently screened the literature and extracted information. The Prediction Model Risk of Bias Assessment Tool (PROBAST) was used to assess the risk of bias and applicability of the included studies. R 4.5.1 software was used to perform meta-analyses of the area under the curve (AUC) values and predictive factors of the models.

**Results:**

A total of 25 studies were ultimately included in this study, with sample sizes ranging from 90 to 8,878 cases and PHN incidence rates ranging from 6.2% to 52.9%. Among them, 18 studies performed internal validation and 4 studies performed external validation. The literature quality assessment results indicated high risk of bias and good applicability in all studies. The area under the receiver operating characteristic curve (AUC) of the models ranged from 0.71 to 0.98. Meta-analysis results showed that the pooled AUC was 0.86 (0.82–0.90), indicating good predictive performance. In addition, Age, VAS, rash site, Prodromal pain, and Extent of Rash were common predictive factors for the occurrence of postherpetic neuralgia.

**Conclusion:**

Research on risk prediction models for postherpetic neuralgia is still at an early stage, with an overall high risk of bias and a lack of clinical application. In the future, scholars may develop high-quality risk prediction models with high accuracy and strong generalizability based on machine learning methods and multicenter, large-sample prospective studies.

**Systematic review registration:**

https://www.crd.york.ac.uk/PROSPERO/view/CRD420261354649, Identifier CRD420261354649

## 1 Introduction

Herpes zoster (HZ) is a common viral disease caused by reactivation of the varicella-zoster virus (VZV), and its clinical manifestations are characterized by a painful rash distributed unilaterally along dermatomes (1). With the intensification of global population aging, the incidence of HZ has shown a marked upward trend. Aging is accompanied by immunosenescence, characterized by a gradual decline in immune function, especially VZV-specific cell-mediated immunity, which plays a critical role in suppressing latent varicella-zoster virus reactivation. Consequently, older individuals are more susceptible to HZ and subsequent complications such as postherpetic neuralgia (2). Postherpetic neuralgia (PHN), as one of its most common and most severe complications, is usually defined as neuropathic pain that persists for more than 3 months after rash healing and affects approximately 10%–30% of patients with HZ (3). The pathogenesis of PHN is complex and involves sensitization of peripheral neurons and the central nervous system. The inflammatory response induced by VZV activation can lead to damage to nerve fibers, thereby triggering abnormal neuronal impulse discharges (4). PHN can cause persistent or paroxysmal pain, sensory abnormalities, sleep disorders, and emotional problems, severely affecting patients’ quality of life and imposing a heavy economic burden on the healthcare system (5). Epidemiological studies have shown that approximately 9%–14% of patients with herpes zoster still experience persistent pain 1 month after disease onset, while approximately 5% still have significant pain symptoms after 3 months (6). Because PHN is characterized by severe pain and prolonged duration, patients are often accompanied by serious sleep disturbances, anxiety, and depression, and may even develop suicidal tendencies. Although antiviral drugs, neuromodulatory agents, and minimally invasive interventional treatments are widely used in clinical practice, pain relief remains unsatisfactory in approximately 30–50% of patients (7). Although antiviral therapies can reduce viral replication and acute inflammation, they may not completely prevent nerve injury or the development of persistent neuropathic pain once neural damage has occurred (8). This “refractoriness” in treatment highlights the importance of early prevention and risk stratification.

Therefore, early identification of populations at high risk for PHN and implementation of interventions are key to reducing the incidence of PHN. Systematic comprehensive evaluations of risk factors have found that clinical characteristics such as age, severity of acute pain, extent of rash, and involvement of specific sites are closely associated with the occurrence of PHN (9, 10). In addition, anxiety/depression, chronic obstructive pulmonary disease (COPD), hypertension, diabetes, and other chronic diseases may also increase the risk of PHN, providing more potential indicators for precise risk assessment (11, 12). Although multiple studies have explored risk factors for PHN, the predictive ability of a single factor is limited. Therefore, researchers have attempted to construct risk prediction models to integrate multiple variables and improve predictive accuracy.

Risk prediction models can comprehensively analyze multiple risk factors for disease and assign corresponding weights to each factor, thereby calculating the probability or risk of future disease occurrence and providing decision-making references for healthcare professionals (13–15). This facilitates the sharing of medical information, helps healthcare professionals make informed medical decisions, promotes the implementation of preventive measures, and encourages changes in patient behavior, ultimately reducing the likelihood of disease occurrence (16). In recent years, with the development of evidence-based medicine and data analysis methods, an increasing number of studies have attempted to establish PHN risk prediction models, with the aim of quantitatively evaluating the probability of PHN occurrence by integrating variables such as demographic characteristics, clinical manifestations, and comorbidities, thereby guiding individualized clinical treatment and prevention strategies (17, 18). However, the currently published PHN prediction models differ greatly in terms of study design, sample sources, predictor selection, and model evaluation methods, and their predictive performance and clinical applicability still require further systematic evaluation (17, 19, 20).

Therefore, systematically reviewing and comprehensively evaluating existing PHN risk prediction models is of great significance for clarifying model construction methods, comparing predictive performance, and guiding future research. This study intends to summarize and analyze the research characteristics, predictors, and model performance of currently published PHN risk prediction models through systematic review and meta-analysis, in order to provide references for clinical risk assessment and future model optimization.

## 2 Materials and methods

### 2.1 Literature inclusion and exclusion criteria

*Inclusion criteria*: (1) study participants aged ≥18 years; (2) patients diagnosed with herpes zoster; (3) the research topic of the literature was the construction and validation of a risk prediction model for postherpetic neuralgia; (4) study types included cohort studies, cross-sectional studies, and case–control studies; and (5) the publication language was Chinese or English.

This systematic review were conducted following the Preferred Reporting Items for Systematic Reviews and Meta-Analyses (PRISMA) statement, a recognized guideline for such research. This systematic review was registered on PROSPERO. Number: CRD420261354649.

*Exclusion criteria*: (1) studies that only analyzed risk factors but did not establish a risk prediction model; (2) incomplete data or inability to obtain the full text; (3) duplicate publications; (4) conference papers; (5) studies that constructed models based on the results of systematic reviews or meta-analyses; and (6) models with fewer than 2 predictive variables.

### 2.2 Literature search strategy

We systematically searched PubMed, Web of Science, Embase, the Cochrane Library, China National Knowledge Infrastructure, Wanfang Database, China Biology Medicine disc (CBM), and VIP Chinese Science and Technology Journal Full-text Database for studies on the establishment and validation of risk prediction models for the occurrence of postherpetic neuralgia after herpes zoster. The search period was limited from database inception to March 1, 2026, and the search languages were Chinese or English. The search process combined subject headings and free-text terms and was supplemented by manual searches to trace additional references. The specific search terms included: *postherpetic neuralgia, herpes zoster postherpetic neuralgia, PHN, prediction model, risk prediction model, risk prediction, risk factors,* and *risk assessment*.

In this systematic review, we adopted the PICOTS framework recommended by the *Checklist for Critical Appraisal and Data Extraction for Systematic Reviews of Prediction Modelling Studies* (CHARMS). This framework helps clarify the review objectives, search strategy, and study inclusion and exclusion criteria:

P (Population): Patients clinically diagnosed with HZ or those with a disease code of HZ.

I (Index prognostic model): Risk prediction model for postherpetic neuralgia.

C (Comparative model): No competing model.

O (Outcome): Occurrence of postherpetic neuralgia in patients with herpes zoster.

T (Timing): Basic information and laboratory indicators of patients with herpes zoster were assessed to predict whether they would develop postherpetic neuralgia.

S (Setting): The risk prediction model can predict the likelihood of postherpetic neuralgia based on the individual conditions of patients with herpes zoster, helping to formulate and implement interventions and avoid adverse events.

### 2.3 Literature screening and data extraction methods

First, NoteExpress was used to remove duplicate records from the retrieved literature. Then, two researchers who had received training in evidence-based courses independently read the literature and excluded duplicate publications based on titles and abstracts. Screening was performed according to the literature inclusion and exclusion criteria, followed by cross-checking. In the case of disagreement, a third researcher was consulted for adjudication to determine the final included literature.

The Checklist for Critical Appraisal and Data Extraction for Systematic Reviews of Prediction Modelling Studies (CHARMS) was first proposed in 2014 by scholars such as Douglas G. Altman from the University of Oxford and Karel G. M. Moons from Utrecht University in the Netherlands to help clarify the questions of systematic reviews and analyze the methodology and quality of original studies (16). Researchers developed a data extraction form based on the CHARMS checklist to extract the characteristics of the literature, including first author, study type, publication year, data source period, modeling method, validation method, predictors, predictive performance, and so on (21). Two researchers independently extracted information from the literature, and when disagreement arose, a third researcher was consulted to assist in judgment and determine the basic information and characteristics of the included literature.

### 2.4 Risk of Bias and applicability evaluation

The Prediction Model Risk of Bias Assessment Tool (PROBAST), developed by Wolff et al. in 2019, is an assessment tool specifically used to evaluate studies developing or validating multivariable diagnostic or prognostic prediction models (22). The risk of bias assessment includes four domains: participants, predictors, outcome, and statistical analysis. Each domain contains 2–9 signaling questions, and each question is answered as “Yes/Probably Yes,” “No/Probably No,” or “No Information,” corresponding to the evaluation results of “low risk of bias,” “high risk of bias,” or “unclear.” If all questions in a domain are rated as “low risk of bias,” then the domain is judged as “low risk of bias.” If one or more questions are rated as “high risk of bias” or “unclear,” then that domain is judged as “high risk of bias” or “unclear.” Applicability assessment includes three domains: participants, predictors, and outcome, without signaling questions, and the evaluation results are similar to those for risk of bias (23, 24). Two researchers used the PROBAST tool to assess the risk of bias and applicability of the included prediction model studies, and a third researcher was consulted when disagreements occurred.

### 2.5 Statistical analysis

In this study, R 4.5.1 software was used for meta-analysis. The area under the receiver operating characteristic curve (AUC) of model predictive performance was used as the effect size indicator, and its specific value and 95% confidence interval (CI) were reported. An AUC between 0.7 and 0.9 indicates moderate predictive validity of the model, whereas an AUC > 0.9 indicates high diagnostic validity. In statistical testing, *p* < 0.05 was regarded as the criterion for statistical significance. Heterogeneity was assessed using Higgins’ I^2^ statistic. When I^2^ ≤ 25%, heterogeneity was considered low; when 25% < I^2^ ≤ 50%, heterogeneity was considered moderate; and when I^2^ > 50%, heterogeneity was considered high. If *p* > 0.05 and I^2^ ≤ 50%, heterogeneity was considered acceptable and a fixed-effects model was used; otherwise, a random-effects model was used to pool effect sizes. Begg’s test, Egger’s test, and funnel plots were used to detect publication bias. *p* < 0.05 was considered statistically significant.

## 3 Results

### 3.1 Literature search process and results

**Figure 1:**
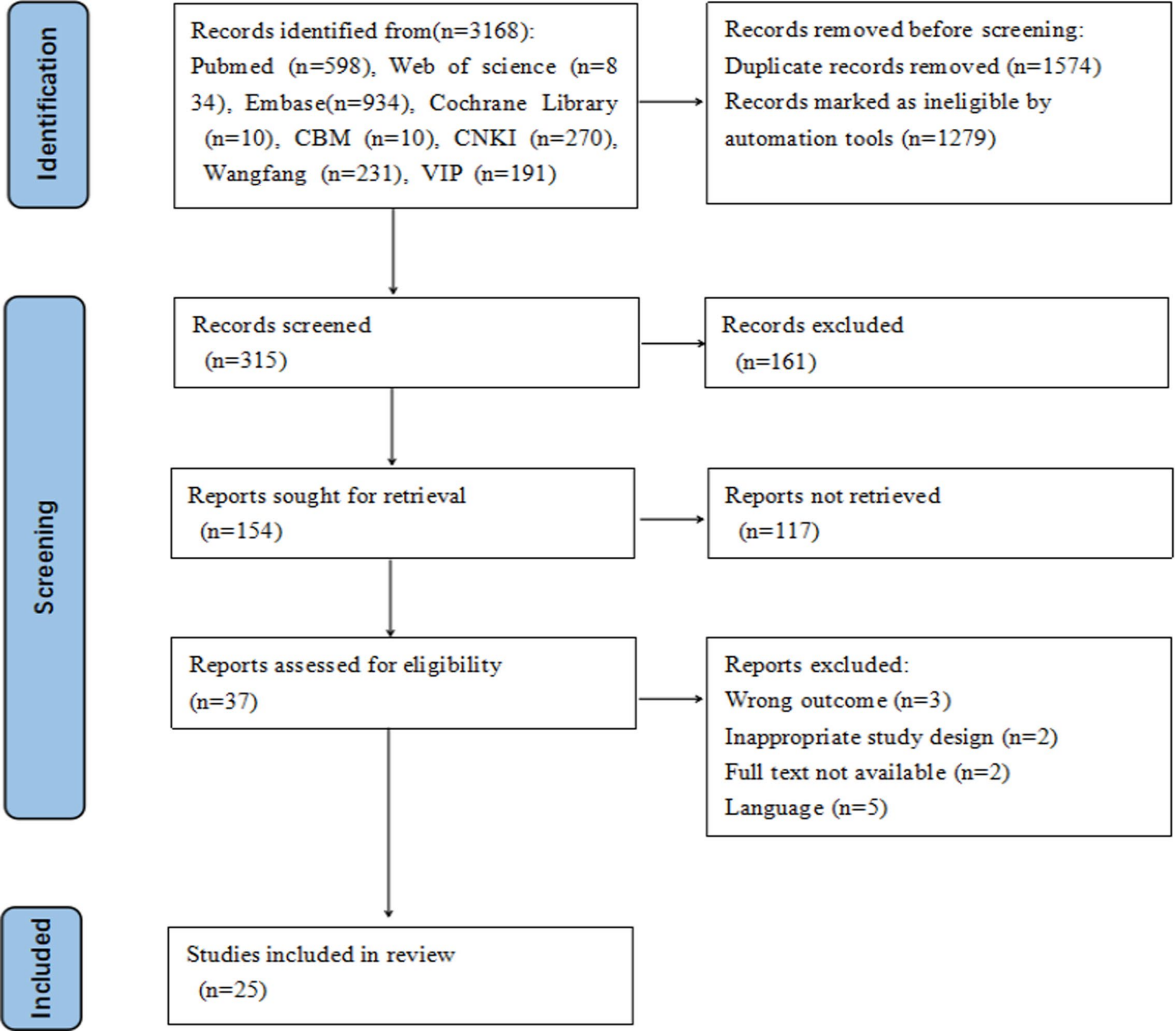
PRISMA flow diagram.

A total of 25 studies were ultimately included in this systematic review after literature identification, duplicate removal, title and abstract screening, and full-text eligibility assessment. The literature screening process is shown in Figure 1.

### 3.2 Basic characteristics of included literature

A total of 25 studies (25–49) were included, most of which were retrospective cohort studies conducted in China. Sample sizes varied considerably across studies. The basic characteristics of the included studies are shown in Table 1.

**Table 1:** Basic characteristics of the included studies.

Study	Year	Country	Study site	Design	Sample
Opstelten et al. (25)	2007	Netherlands	Multiple regions in the Netherlands	Prospective cohort study	598
Cho et al. (26)	2014	Korea	Kangnam sacred heart hospital	Prospective cohort study	305
Wang et al. (27)	2020	China	China-japan friendship hospital	Retrospective cohort study	502
Kinouchi et al. (29)	2021	Japan	Fukagawa hospitals, asahikawa hospitals	Retrospective cohort study	761
Wang et al. (28)	2021	China	Beijing university of Chinese medicine	Retrospective cohort study	706
Zhang et al. (33)	2022	China	Beijing friendship hospital	Retrospective cohort study	732
Zhou et al. (34)	2022	China	West china hospital	Case–control study	60
Liu et al. (31)	2022	China	China-japan friendship hospital	Prospective cohort study	174
Li et al. (30)	2022	China	Mianyang central hospital	Retrospective cohort study	425
Lu et al. (32)	2022	China	Guangdong medical university	Retrospective cohort study	150
Mao et al. (35)	2023	China	Xiaogan hospital	Retrospective cohort study	272
Lu et al. (39)	2023	China	Jinshan hospital	Prospective cohort study	90
Tian et al. (36)	2023	China	Nanxishan hospital	Retrospective cohort study	452
Zhao (38)	2023	China	Jining tertiary hospital	Retrospective cohort study	889
Wang et al. (37)	2023	China	Shandong skin hospital	Retrospective cohort study	307
Fan (43)	2024	China	China-japan friendship hospital	Prospective cohort study	174
Lin et al. (41)	2024	China	Zhejiang tcm hospital	Retrospective cohort study	524
Wang et al. (42)	2024	China	Northern jiangsu hospital	Retrospective cohort study	2,420
Yang et al. (40)	2024	China	Xijing hospital, 940 hospital	Retrospective cohort study	481
Park et al. (49)	2025	Korea	Clinical data warehouse	Retrospective cohort study	8,878
Liu et al. (46)	2025	China	Jingdezhen dermatology hospital	Retrospective cohort study	230
Luo et al. (47)	2025	China	Suining central hospital	Retrospective cohort study	480
Lin et al. (45)	2025	China	Zhejiang hospital	Retrospective cohort study	329
Fei et al. (44)	2025	China	Zhoupu hospital	Retrospective cohort study	572
Zhen et al. (48)	2025	China	Suining central hospital	Retrospective cohort study	200

### 3.3 Methods for model construction and predictive performance inclusion

Among the 25 included studies (25–49), Logistic regression was the most commonly used modeling method, while several studies additionally applied machine learning algorithms such as random forest, support vector machine, XGBoost, and neural networks. Most models demonstrated acceptable to excellent predictive performance, with reported AUC values generally exceeding 0.75. Detailed information on model construction methods and predictive efficacy is shown in Tables 2, 3.

**Table 2:** Construction methods of the included prediction models.

Study	Modeling dataset	Validation dataset	Incidence	Validation method	Variable selection	Modeling method	Performance evaluation
Opstelten et al. (25)	598	—	7.7%	—	C, D	F	S
Cho et al. (26)	305	—	6.2%	—	D	F	—
Wang et al. (27)	502	502/60	24.9%	A, B	C, D	F, G*	T
Kinouchi et al. (29)	502	502/259	15.4%	A, B	C, D	H	—
Wang et al. (28)	706	204	—	A	C, D	F, G*, I	—
Zhang et al. (33)	600	132	19.4%	A	E	J, F	U
Zhou et al. (34)	60	—	—	—	E	F, G*, J	U
Liu et al. (31)	174	174	29.9%	A	C, D	F	S
Li et al. (30)	425	425	30.1%	A	C, D	F	S
Lu et al. (32)	150	150	37.3%	A	C, D	K	S
Mao et al. (35)	258	—	32.2%	—	C, D	F	—
Lu et al. (39)	90	—	—	—	D	F	—
Tian et al. (36)	226	190	17.6%	A	C, D	F	T
Zhao (38)	622	267/161	30.60%	A, B	C, D	F, G, L*	T
Wang et al. (37)	307	307	32.8%	A	D, E	F	S
Fan (43)	174	—	29.9%	—	D	F	S
Lin et al. (41)	419	105	43.7%	A	E	F, J, G*, M, N, O	T
Wang et al. (42)	1,696	724	16.3%	A	C, D, E	F	T
Yang et al. (40)	434	434	45.4%	A	E	F, J, L*	U
Park et al. (49)	8,878	8,878	9.0%	A	C, D	F, G, J, L*, P	U
Liu et al. (46)	160	160/70	28.1%	A, B	C, D	F	T
Luo et al. (47)	240	240	21.7%	A	C, D	F	T
Lin et al. (45)	329	—	52.9%	—	E	F, L, J, G*, Q, H, O, R	—
Fei et al. (44)	400	172	25.9%	A	D	G*, F, J	U
Zhen et al. (48)	140	140/60	30.71%	A, B	C, D	F	T

A, internal validation; B, external validation; C, univariate analysis; D, multivariate analysis; E, Lasso regression; F, logistic regression; G, random forest; H, Naive Bayes; I, artificial neural network; J, support vector machine; K, Cox regression; L, Xgboost; M, K Nearest Neighbor; N, gradient boosting; O, neural network; P, multilayer perceptron; Q, decision tree; R, gradient boosting; S, bootstrapping; T, random split validation; U, cross-validation; *, optimal model.

**Table 3:** Predictive performance of the included prediction models.

Study	Validation method	AUC	95%Cl	Calibration
Opstelten et al. (25)	–	0.77	0.71–0.82	H-L test (*p* = 0.49)
Cho et al. (26)	Sensitivity = 78.0%, Specificity = 78.9%	0.87	—	—
Wang et al. (27)	Sensitivity = 94.0%, Specificity = 97.0%	0.98	0.96–0.99	—
Kinouchi et al. (29)	—	0.76	0.63–0.86	—
Wang et al. (28)	—	0.88	—	—
Zhang et al. (33)	Sensitivity = 84.0%, Specificity = 87.9%	0.88	—	—
Zhou et al. (34)	—	0.96	—	—
Liu et al. (31)	—	0.81	0.77–0.95	Calibration curve
Li et al. (30)	—	0.81	0.79–0.84	Calibration curve
Lu et al. (32)	—	0.77	0.67–0.93	Calibration curve
Mao et al. (35)	—	0.90	0.85–0.94	Calibration curve
Lu et al. (39)	—	0.85	0.76–0.93	—
Tian et al. (36)	—	0.79	0.73–0.85	Calibration curve
Zhao (38)	Sensitivity = 86.6%, Specificity = 65.5%	0.86	0.83–0.89	Calibration curve
Wang et al. (37)	Sensitivity = 69.3%, Specificity = 82.5%	0. 83	0.78–0.88	Calibration curve, H-L test (*p* = 0.168)
Fan (43)	—	0.81	0.77–0.95	Calibration curve
Lin et al. (41)	Specificity = 97.1%	0.93	0.88–0.98	Calibration curve
Wang et al. (42)	—	0.71	0.66–0.76	Calibration curve, H-L test
Yang et al. (40)	Sensitivity = 92.0%, Specificity = 86.0%	0.86	0.79–0.94	Calibration curve, H-L test (*P* = 0. 162)
Park et al. (49)	Sensitivity = 30.8%, Specificity = 96.0%	0.79	—	—
Liu et al. (46)	Sensitivity = 73.7%, Specificity = 96.1%	0.91	0.86–0.99	H-L test (*p* = 0.525)
Luo et al. (47)	—	0. 97	0. 96–0.99	H-L test (*p* = 0.933)
Lin et al. (45)	—	0.88	0.82–0.93	—
Fei et al. (44)	Sensitivity = 86.4%, Specificity = 92.2%	0.88	—	Calibration curve
Zhen et al. (48)	Sensitivity = 94.7%, Specificity = 92.7%	0.98	0.95–1.00	Calibration curve, H-L test (*p* = 0.911)

### 3.4 The predictive factors included in the models

Among the 25 studies (25–49) included in this study, Age, VAS, Rash Site, Prodromal Pain, and Extent Of Rash were the most common predictive factors. The predictive factors of the specific models are shown in Table 4.

**Table 4:** Predictive factors of the risk prediction models.

Study	Predictive factors
Opstelten et al. (25)	Age, VAS, extent of rash
Cho et al. (26)	S-LANSS, VAS, age
Wang et al. (27)	Age, NRS, rash site, CCI, antiviral therapy, immunosuppression
Kinouchi et al. (29)	Age, immunosuppression, rash severity, VAS
Wang et al. (28)	Age, rash site, NRS, CCI, Antiviral therapy, immunosuppression
Zhang et al. (33)	Age, VAS, rash site, antiviral therapy delay
Zhou et al. (34)	Plasma metabolites, proteins
Liu et al. (31)	sex, age, prodromal pain, extent of rash, VAS
Li et al. (30)	Age, diabetes, smoking, extent of rash, VAS, abnormal CD4+/CD8 + ratio
Lu et al. (32)	Age, Diabetes mellitus, Prodromal pain, extent of rash, VAS, Initial treatment time
Mao et al. (35)	Age, delayed treatment, rash site, statin use, underlying disease, NSE, TG, VAS
Lu et al. (39)	Metabolomic markers related to varicella-zoster infection
Tian et al. (36)	Age, Prodromal pain, delayed treatment, CRP
Zhao (38)	Age, Coronary heart disease, Severe skin lesions, Moderate to severe pain during hospitalization
Wang et al. (37)	Age, Diabetes mellitus, extent of rash, Prodromal pain, NRS
Fan (43)	Age, VAS, extent of rash, delayed treatment
Lin et al. (41)	Age, prodromal pain, lesion distribution, immune status
Wang et al. (42)	Age, VAS, rash site, antiviral therapy timing
Yang et al. (40)	Age, VAS, rash site, affected dermatome, timing of nerve block treatment, pain characteristics
Park et al. (49)	Age, sex, immunosuppression, rash site, antiviral therapy timing
Liu et al. (46)	Age, PHQ-9, PSQI, VAS, HADS
Luo et al. (47)	Sex, prodromal pain, diabetes, rash site, delayed treatment
Lin et al. (45)	Education level, marital status, dietary habits, lesion duration, rash site, lesion sequence, prodromal pain
Fei et al. (44)	Age, sex, prodromal pain, VAS, rash characteristics, time to treatment
Zhen et al. (48)	Age, Th1 level, Th2 level, Th17 level, CRP, Neu

VAS, Visual Analog Scale; S-LANSS, The Self-completed Leeds Assessment of Neuropathic Symptoms and Signs pain scale; NRS, Numeric Rating Scale; CCI, Charlson Comorbidity Index; TG, triglyceride; NSE, Neuron Specific Enolase; CRP, C-reactive protein; PHQ-9, Patient Health Questionnaire-9; PSQI, Pittsburgh Sleep Quality Index; HADS, Hospital Anxiety Depression Scale; Neu, Neutrophil count.

### 3.5 The methodological quality assessment results of the included studies

#### 3.5.1 Study population domain

The bias related to study participants mainly arose from study type. Among all the included studies, 19 studies (27–30, 32, 33, 35–38, 40–42, 44–49) were retrospective cohort studies and 1 study (34) was a case–control study; therefore, they were judged to be at high risk of bias.

#### 3.5.2 Predictor domain

In the 25 included studies (25–49), the standards used for different study participants were consistent in terms of definitions and assessment methods, and the evaluation criteria were clearly specified. When assessing predictors, the researchers used blinding to ensure the confidentiality of outcome information. In addition, all predictors in the included studies were statistically significant, so all were judged to be at low risk of bias.

#### 3.5.3 Outcome domain

When defining outcome variables, all 25 included studies (25–49) used recognized methods such as guidelines or consensus statements, and the time interval between the measurement of predictors and outcome indicators was established based on clinical expertise. Therefore, the bias related to outcomes was low.

#### 3.5.4 Statistical analysis domain

The included studies showed a generally high risk of bias in the statistical analysis domain. Common methodological limitations included insufficient sample size, inadequate reporting of missing data handling, lack of external validation, reliance on single-center data sources, absence of calibration assessment, and the use of random split-sample validation methods. A summary of the major sources of statistical analysis bias is presented in Table 5.

**Table 5:** Summary of sources of statistical analysis bias in included studies.

Source of bias	Number of studies	Studies
Insufficient sample size	8	(31, 32, 34, 35, 39, 43, 46, 48)
Missing data handling not reported	5	(25, 28, 39, 40, 46)
Single-center design	21	(26–28, 30–39, 41–48)
External validation performed	4	(29, 38, 46, 48)
Calibration not reported	9	(26–29, 33, 34, 39, 45, 49)
Random split-sample validation used	8	(28, 36, 38, 41, 42, 45, 47, 48)

#### 3.5.5 Applicability assessment

**Figure 2:**
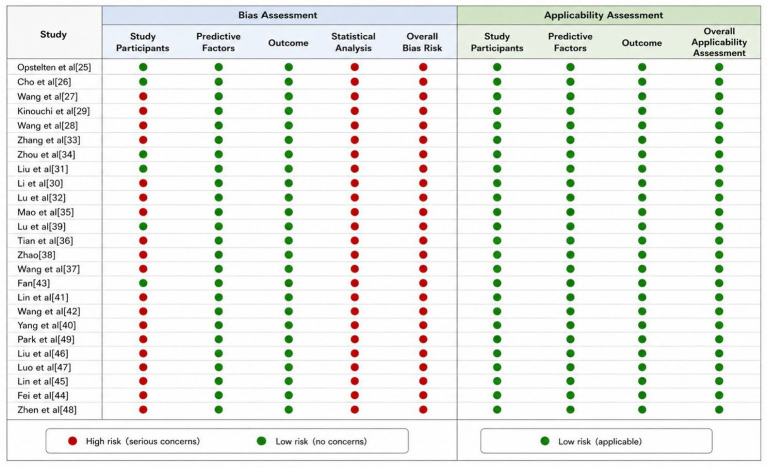
Risk of bias and applicability assessment of included prediction models using the PROBAST tool.

Applicability evaluation was conducted for all included studies, including three aspects: study participants, predictors, and outcomes. The results showed that all included models had good applicability in these three aspects, and the overall level of applicability assessment was high. The bias and applicability assessments of the included models are shown in Figure 2.

### 3.6 Meta-analysis results

**Figure 3:**
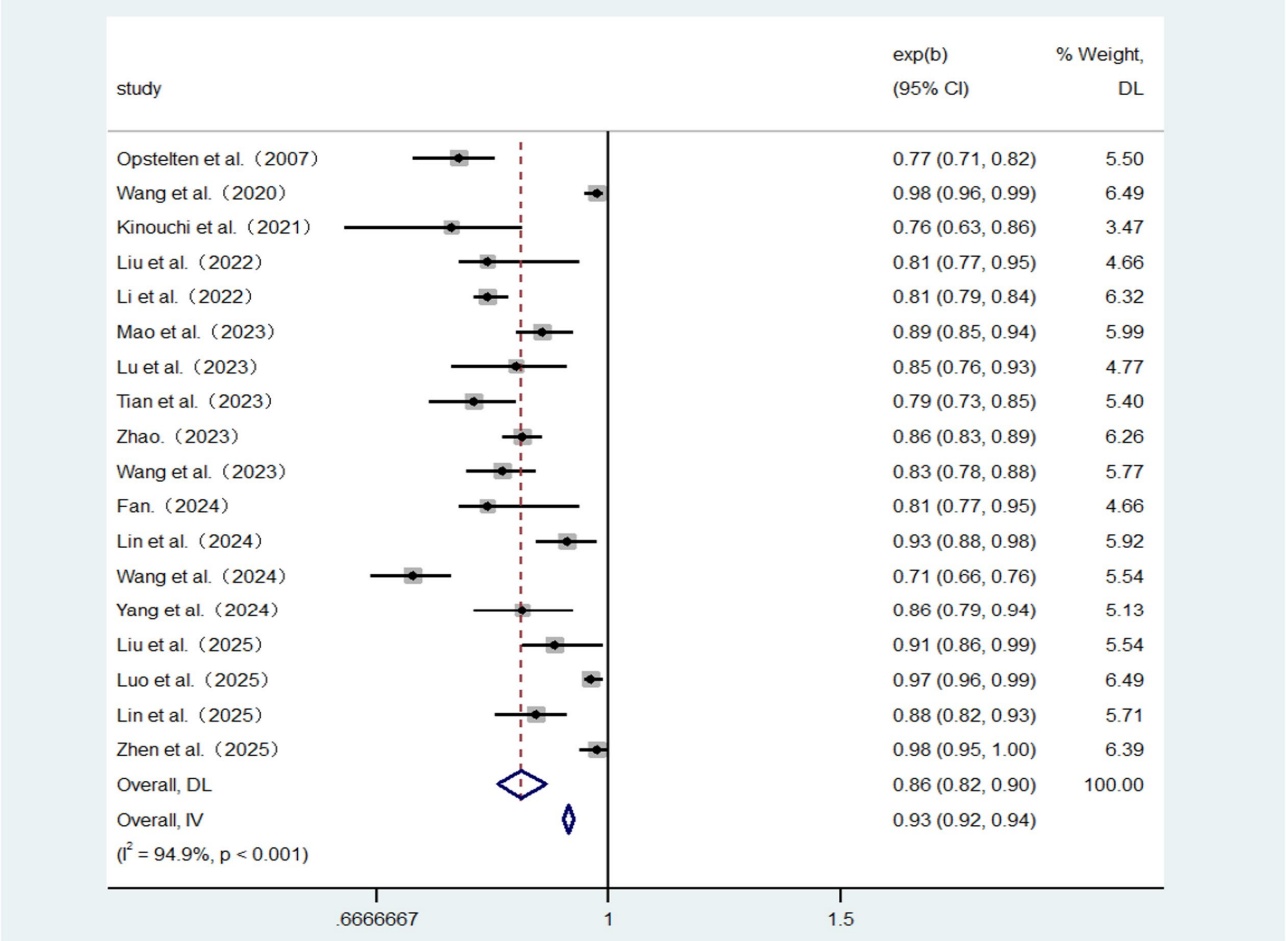
Forest plot of the area under the receiver operating characteristic curve for the risk prediction model.

Eighteen studies (25, 27, 29–32, 35–38, 40–43, 45–48) reporting AUC values and 95% confidence intervals were included in the meta-analysis. The results showed heterogeneity (*I*^2^ = 94.9%, *p* < 0.001); therefore, a random-effects model was selected to pool the effect sizes. A random-effects model was applied when substantial heterogeneity was present (*I*^2^ > 50%). In addition, considering the potential clinical and methodological heterogeneity among the included studies, including differences in study populations, predictor selection, model development methods, and validation strategies, the random-effects model was considered more appropriate to provide conservative pooled estimates. The pooled AUC was 0.86 (0.82–0.90), indicating good predictive performance. The forest plot of the pooled AUC is shown in Figure 3.

**Figure 4:**
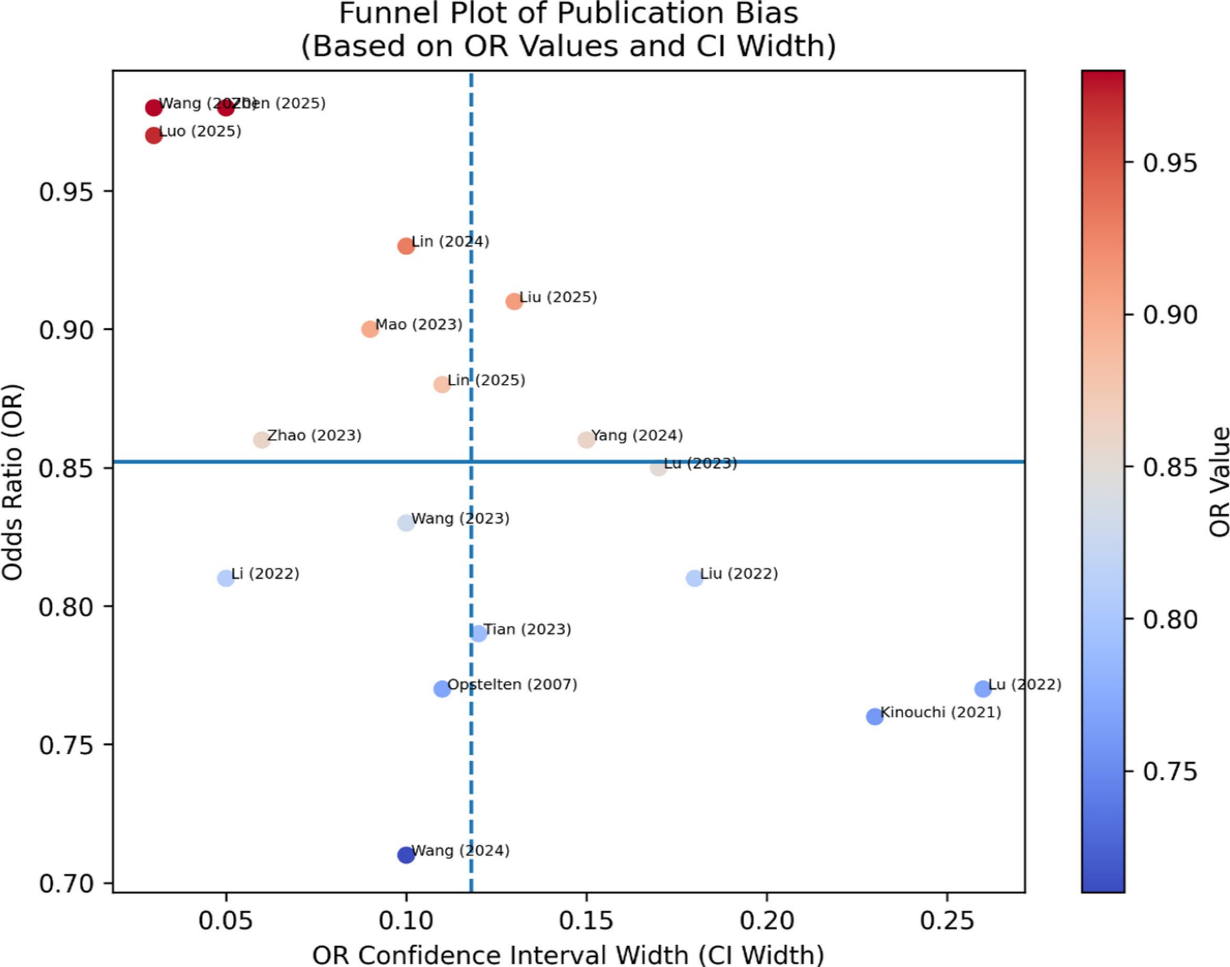
Funnel plot examination.

A funnel plot was drawn to detect publication bias based on the model AUCs in the included studies. The results showed that the studies were symmetrically distributed on both sides, indicating no obvious publication bias. The funnel plot is shown in Figure 4. In addition, Begg’s test showed Z = −2.069, *p* = 0.71; Egger’s test showed t = −1.34, *p* = 0.63, confirming the absence of publication bias.

## 4 Discussion

### 4.1 Age, VAS, rash site, prodromal pain, extent of rash are common predictors

The results of this study found that Age, VAS, rash site, Prodromal pain, and extent of rash are common predictors.

Twenty one studies considered Age to be a predictor of postherpetic neuralgia. This is consistent with the findings of Zhou et al. (50). With increasing age, the body’s cellular immune function gradually declines, especially the T cell-mediated immune response, making varicella-zoster virus more likely to reactivate and cause nerve damage. At the same time, the nerve regeneration ability of the elderly is weakened and pain regulation ability declines, making them more likely to develop chronic neuropathic pain (51). Therefore, medical staff should regard elderly HZ patients as the key population for PHN prevention, strengthen pain monitoring, standardize antiviral treatment and health education in the early stage of onset, and arrange closer follow-up after discharge to identify pain prolongation trends as early as possible. Existing studies clearly indicate that PHN risk increases with age and is more prominent in middle-aged and elderly patients.

Eighteen studies considered VAS to be a predictor of postherpetic neuralgia. This is consistent with the findings of Forbes et al. (52). This may be because acute-phase pain is caused by skin damage and peripheral nerve inflammatory response, and pain intensity may represent the severity of nerve damage. The more severe the nerve damage, the longer it takes for the body to repair, and the nerve repair process is also the process of neural remodeling changes leading to central sensitization, thereby easily leading to PHN (53). Therefore, medical staff should pay attention to the early assessment and intervention of acute-phase pain and adopt standardized analgesia protocols to reduce the risk of pain chronification.

Ten studies considered Rash Site to be a predictor of postherpetic neuralgia. Previous literature suggests that certain special affected sites, especially the head and face, ophthalmic branch, or areas with dense sensory nerve distribution, are often associated with higher PHN incidence risk (54). The reason may be that these areas have dense nerve distribution, more severe sensory nerve damage, and nerve damage in the face and head is more likely to lead to central sensitization and pain chronification (55). Therefore, medical staff should carefully record the rash distribution sites and affected dermatomes in clinical work, raise vigilance for patients with involvement of the head and face, periorbital area, or pain-sensitive areas, and, if necessary, initiate early multidisciplinary management involving pain, dermatology, or ophthalmology departments to reduce the risk of complications and pain prolongation.

Eight studies considered Prodromal Pain to be a predictor of postherpetic neuralgia. Existing studies have shown that Prodromal Pain is an independent influencing factor for PHN (56). Prodromal Pain often occurs before rash formation, suggesting that before clinically visible skin lesions appear, the virus has already caused inflammatory response and damage to the sensory ganglia and peripheral nerves. This means that patients may have significant nerve involvement in the early stage of the disease, and therefore have a higher probability of subsequent persistent neuropathic pain. The presence of prodromal pain may also reflect greater sensitivity of the body to pain stimuli, suggesting a stronger tendency for pain chronification (57). Based on this, medical staff should proactively inquire about the presence of pre-rash pain, duration, and nature when seeing HZ patients, and should not only focus on the rash itself. Once obvious prodromal pain is found, it should be included in the high-risk assessment scope, and pain management and health education should be initiated early.

Six studies considered Extent of Rash to be a predictor of postherpetic neuralgia. This may be because a larger rash area indicates more active viral replication, stronger local and segmental inflammatory response, and involvement of more nerve fibers, thereby leading to more severe nerve damage and more persistent abnormal pain signal transmission. In addition, a wider range of rashes is usually accompanied by more severe acute-phase symptoms, also indicating a heavier disease burden (58). Medical staff should pay attention to recording rash extent, herpes density, and severity during routine physical examination and use it as an important component of risk assessment. In addition, for patients with extensive skin lesions, medical staff should strengthen early antiviral treatment, pain control, and follow-up management to minimize further progression of nerve damage in the acute phase. Regarding the relationship between severe rash or larger rash area and increased PHN risk.

### 4.2 Model predictive ability is good, but bias still exists

The meta-analysis results of this study showed that the pooled AUC of the included models was 0.86 (0.82–0.90), suggesting that the existing PHN risk prediction models generally have good discrimination. In addition, by integrating information such as age, pain severity, rash extent, prodromal pain, and underlying diseases, the models can identify high-risk populations for PHN to a certain extent and provide a basis for early clinical intervention. However, this study still has certain biases in multiple aspects.

In terms of study type, among the literature included in this study, 19 studies (27–30, 32, 33, 35–38, 40–42, 44–49) were retrospective cohort studies, 1 study (34) was a case–control study, and 5 studies (25, 26, 31, 39, 43) were prospective cohort studies. Retrospective studies are difficult to accurately quantify risk or predict future events due to time confounding, recall bias, and lack of dynamic data. Risk prediction models constructed based on prospective studies can actively confirm outcomes through standardized follow-up procedures, clarify the temporal relationship between predictors and outcomes, and ultimately produce a predictive tool that can be robustly applied in the real world (59).

In terms of sample size, 8 studies (31, 32, 34, 35, 39, 43, 46, 48) had insufficient sample sizes in the model development and validation stages. Sample size is usually determined by the number of events per variable (EPV). It is currently recommended that EPV should be at least >20 to reduce potential bias in the model (60). Adequate sample size can capture more variability and potential confounding factors, improve the precision of results, reduce standard errors, and enhance the generalizability of the model (60). At the same time, 21 studies (26–28, 30–39, 41–48) were single-center studies, and only 4 studies (29, 38, 46, 48) conducted external validation, which limits the generalizability of the models. Multicenter studies, especially those conducted in multiple regions, can increase the diversity and representativeness of samples, reduce inherent biases in single-center studies, and improve the generalizability and extrapolability of research results (61). Therefore, future scholars are recommended to conduct large-sample multicenter studies.

In terms of variable selection, only 7 studies (33, 34, 37, 40–42, 45) used LASSO regression models to screen variables, which may increase the risk of selecting erroneous predictors. Variable screening based on LASSO regression or machine learning methods can automatically screen variables, reduce bias risk in statistical analysis, and improve the accuracy and practicality of prediction models (62).

In terms of modeling methods, 23 studies (25–28, 30, 31, 33–49) used logistic regression. Logistic regression models are more robust when the sample size is small and the relationships between variables are linear, and they have stronger interpretability, making them suitable for scenarios requiring clinical interpretability (63). However, they have limitations when dealing with complex multidimensional data. In contrast, machine learning is better at identifying nonlinear relationships and complex interactions between variables, especially when handling high-dimensional data, and performs better in sensitivity, specificity, and predictive effect (64). The continuous advancement of machine learning technology provides strong support for the development and validation of complex models. Future scholars can consider using machine learning methods to construct risk prediction models with good predictive effect and strong stability based on large samples.

In terms of model validation, 18 studies (27–33, 36–38, 40–42, 44, 46–49) conducted internal validation, and only 4 studies (29, 38, 46, 48) conducted external validation. Internal and external validation of models can help ensure the applicability and validity of the models and reduce overfitting (65). It is worth mentioning that external validation can assess the generalization ability and robustness of the model in different populations and environments, thereby improving the credibility and practical application value of the model. In addition, external validation can reveal the performance of the model on new datasets, help identify potential overfitting and bias issues, and thus promote further optimization and improvement of the model (66). In addition, 8 studies (28, 36, 38, 41, 42, 45, 47, 48) used random split validation, 6 studies (25, 30–32, 37, 43) used bootstrapping, and 5 studies (33, 34, 40, 44, 49) used cross-validation. Simple random data splitting will further reduce the effective sample size used for modeling, and the validation results are also easily affected by random sample fluctuations. In contrast, bootstrap or cross-validation can usually make fuller use of the data and provide more robust internal validation results (67).

In addition, 16 studies (25, 30–32, 35–38, 40–44, 46–48) reported calibration. Calibration, as a key step in risk prediction models, mainly reflects the consistency between the actual observed outcome occurrence frequency and the model-predicted frequency (68). If calibration is not reported, the accuracy of model prediction cannot be evaluated.

### 4.3 Implications for future research

Risk prediction models are one of the hot topics in clinical research in recent years. They can identify high-risk populations through early screening, thereby helping medical staff formulate more targeted prevention and intervention measures, improve the efficiency of medical resource utilization, and improve patient outcomes. This study systematically summarized the current research status of risk prediction models for postherpetic neuralgia and found that although existing models show good predictive performance, there is still room for further optimization in study design, modeling methods, and clinical translation.

In terms of modeling methods, constructing disease risk prediction models based on machine learning algorithms has become an important research direction in the interdisciplinary field of medicine and computer science. Compared with traditional logistic regression, machine learning methods are better at identifying complex nonlinear relationships and high-order interactions between variables (69, 70). With the continuous development of related technologies, they also provide stronger technical support for the development and validation of complex models (71, 72). Existing studies have shown that models established with machine learning algorithms often have better predictive performance than logistic regression models (73). Therefore, future research can further explore the construction of high-performance PHN risk prediction models based on machine learning algorithms to improve the discrimination ability and clinical application value of the models.

In terms of model presentation, future models can be transformed into regression equations, nomograms, online risk calculators, and other forms that are convenient for clinical use, and attempts can be made to integrate them with electronic medical record systems to better meet the actual needs of medical staff. This can not only improve prediction efficiency under the support of complex algorithms but also reduce the burden of manual calculation and improve the operability and generalizability of the model in clinical practice.

In terms of predictor screening, there are certain differences in the predictors included in different studies, which may be related to differences in candidate variable sources and heterogeneity of study subjects (74, 75). To further optimize predictor selection, it is recommended that future researchers systematically screen variables with clinical significance, strong accessibility, and high stability by combining literature review, statistical analysis results, clinical guidelines, and expert consensus, thereby improving the scientificity and practicality of the models.

In addition, through the methodological quality evaluation of the included studies, it was found that study participant selection and statistical analysis process were the main reasons for the overall high risk of bias. At the same time, most of the prediction models in the included literature were retrospective studies, and most lacked large-sample external validation, which to a certain extent limits the extrapolability and predictive effect of the models.

Therefore, future research should conduct large-sample, multicenter, prospective studies and strengthen external validation and updating of models to explore and construct more stable, more accurate, and more generalizable optimal prediction models. Medical staff can also use data mining and artificial intelligence technology to build a PHN risk prediction information platform, further improving the accuracy of model prediction and clinical work efficiency, reducing patients’ economic burden, and lowering overall medical costs.

### 4.4 Limitations

(1) This study only included Chinese and English literature, which may lead to the omission of relevant literature in other languages; (2) When evaluating and analyzing the research results, we comprehensively considered all literature but did not consider regional bias, so there may be potential publication bias; (3) We found that most of the included studies were conducted in China, which may introduce publication bias and limit the applicability of the models in other populations, so our research results may not fully represent individuals from different regions and ethnicities. Additionally, differences in ethnicity, healthcare systems, clinical practice patterns, and population characteristics across regions may influence predictor distribution and model performance, thereby affecting the generalizability of these prediction models. Future studies should further validate these models in more diverse populations and multicenter international settings. (4) Among the studies included in this study, only 5 were prospective cohort studies. In addition, only 4 were multicenter studies and 4 conducted external validation, which may affect the stability, extrapolability, and predictive performance of the models.

## 5 Conclusion

Existing prediction models for postherpetic neuralgia generally demonstrated acceptable predictive performance and may help identify high-risk patients for early prevention and intervention. However, considerable heterogeneity was observed among studies in terms of study design, predictor selection, modeling approaches, and validation strategies. In addition, many included studies showed a high risk of bias and lacked adequate external validation, which may limit the reliability and generalizability of the reported model performance. Future research should focus on conducting large-sample, multicenter prospective studies and strengthening external validation in more diverse populations to develop more robust, accurate, and clinically applicable PHN risk prediction models.

## Data Availability

The original contributions presented in the study are included in the article/supplementary material, further inquiries can be directed to the corresponding author.
